# Book Reviews

**Published:** 1897-01

**Authors:** 


### Book Reviews

AN AMERICAN TEXT-BOOK OF PHYSIOLOGY. Edited by W. W. Howell, Ph. D., M. D., Professor of Physiology in the Johns Hopkins University; assisted by ten Professors of Physiology in American Medical Schools. Fully Illustrated. 1,050 pages. Price, cloth $6.00, sheep $7.00. For sale by subscription only. W. B. Saunders, Philadelphia. 1896.

This volume is uniform with the preceding ones of the Saunders American Text-book series and embraces the subject of physiology in the combined work of ten professors in the leading medical schools of the United States. Experimental physiology has been comprehensively dealt with and the salient features of progress in this important branch have been presented. The teacher of physiology will find this volume extremely useful and thorough and the student who wishes to have a higher and clearer knowledge of one of the fundamental structures of medicine than is possible to be gained from the elementary treatises generally used in the medical schools, would do well to have this splendid work in his possession.

BEDSIDE RECORD.

The Imperial Granum Co. have issued convenient blanks for preserving bedside records. The sheets are neatly bound and will be supplied gratis to physicians and trained nurses.

MINOR SURGERY AND BANDAGING. By Henry R. Wharton, M. D., Demonstrator of Surgery in the University of Pennsylvania. New (3d) edition. In one 12mo. volume of 594 pages, with 475 engravings, many being photographic. Cloth, $3. Philadelphia: Lea Brothers & Co., 1896.

Wharton's manual has already achieved for itself an enviable reputation, and this edition, containing the admirable features of the older ones, has, in addition, much new material on the subject of excision of the joints and operations upon the nerves and tendons. The illustrations, a large number of which are photographic, thoroughly cover the text. Formulæ for making various antiseptic solutions are given and the agents used in securing surgical cleanliness are fully considered. The descriptions of the operative procedures are brief and to the point.

A MANUAL OF VENEREAL DISEASES. By Jas. R. Hayden, M. D., chief Venereal Clinic, College of Physicians and Surgeons, New York. In one 12mo. volume of 263 pages, with 47 engravings. Cloth, $1.50. Lea Bros. & Co., Philadelphia and New York. 1896.

This condensed manual offers an excellent working guide for students and practitioners. All unnecessary material has been omitted and as a result the subject of venereal disease is presented in an entirely direct and practical manner. The operative details are admirably clear and simple. The succinctness and brevity of the book, without the omission of any of the essentials, together with its moderate price, make it a desirable addition to the literature of the subject.

---

THE READY REFERENCE HANDBOOK OF DISEASES OF THE SKIN. By George Thomas Jackson, M. D., Professor of Dermatology, Woman's Medical College of the New York Infirmary and in the University of Vermont. New (2d) edition. In one 12mo. volume of 589 pages, with 69 illustrations and a colored plate. Cloth, $2.75. Philadelphia, Lea Bros. & Co., 1896.

The appearance of a second edition of Dr. Jackson's well known work is an evidence of its progressive popularity. The work is the result of a large personal experience and a careful study of dermatological literature. It is arranged somewhat in the order of Brocq's, that is no classification has been attempted, and the diseases have been considered alphabetically. The book is well illustrated throughout. For the general practitioner, who desires a reference book in this branch of medicine, could not be found a safer guide than that offered by Dr. Jackson.

---

INTERNATIONAL CLINICS. A Quarterly of Clinical Lectures on Medicine, Neurology, Surgery Gynecology, Obstetrics, Ophthalmology, Laryngology, Pharyngology. Rhinology, Otology and Dermatology, and specially prepared articles on treatment. By professors and lecturers in the leading medical colleges of the United States, Germany, Austria, France, Great Britain and Canada. Edited by Judson Daland, M. D., University of Pennsylvania, Philadelphia, Instructor in Clinical Medicine and Lecturer on Physical Diagnosis in the University of Pennsylvania; Assistant Physician to the Hospital of the University of Pennsylvania, etc.; Fellow of the College of Physicians of Philadelphia. J. Mitchell Bruce, M. D., F. R. C. P., London, England, Physician to and Lecturer on the Principles and Practice of Medicine at the Charing Cross Hospital. David W. Finlay, M. D, F. R. C. P., Aberdeen, Scotland, Professor of Practice of Medicine in the University of Aberdeen; Physician to, and Lecturer on, Clinical Medicine in the Aberdeen Royal Infirmary, ete. Volume III. Sixth series. 1896. Octavo, pp. xii.—314. Philadelphia: J. B. Lippincott Co. 1896.

